# Digestive enzymes and sphingomyelinase D in spiders without venom (Uloboridae)

**DOI:** 10.1038/s41598-023-29828-x

**Published:** 2023-02-15

**Authors:** Rodrigo Valladão, Oscar Bento Silva Neto, Marcelo de Oliveira Gonzaga, Daniel Carvalho Pimenta, Adriana Rios Lopes

**Affiliations:** 1 Biochemistry Laboratory, Butantan Institute, São Paulo, 05503900 Brazil; 2 Biotechnology Postgraduate Program, University of São Paulo, São Paulo, 05508000 Brazil; 3 Universidade Federal de Uberlândia, Biomedical Sciences Institute, Uberlândia, Minas Gerais 38400902 Brazil

**Keywords:** Hydrolases, Proteomics

## Abstract

Spiders have distinct predatory behaviours selected along Araneae’s evolutionary history but are mainly based on the use of venom for prey paralysis. Uloboridae spiders have lost their venom glands secondarily during evolution. Because of this, they immobilise their prey by extensively wrapping, and digestion starts with the addition of digestive fluid. During the extra-oral digestion, the digestive fluid liquefies both the prey and the AcSp2 spidroins from the web fibres. Despite the efficiency of this process, the cocktail of enzymes involved in digestion in Uloboridae spiders remains unknown. In this study, the protein content in the midgut of *Uloborus* sp. was evaluated through enzymatic, proteomic, and phylogenetic analysis. Hydrolases such as peptidases (endo and exopeptidases: cysteine, serine, and metallopeptidases), carbohydrases (alpha-amylase, chitinase, and alpha-mannosidase), and lipases were biochemically assayed, and 50 proteins (annotated as enzymes, structural proteins, and toxins) were identified, evidencing the identity between the digestive enzymes present in venomous and non-venomous spiders. Even enzymes thought to be unique to venom, including enzymes such as sphingomyelinase D, were found in the digestive system of non-venomous spiders, suggesting a common origin between digestive enzymes and enzymes present in venoms. This is the first characterization of the molecules involved in the digestive process and the midgut protein content of a non-venomous spider.

## Introduction

The success of spiders during evolution is frequently associated with venom^1,2^, web for prey capture and immobilisation^3^, and digestive enzymes for prey degradation. Spiders digest a large amount of food by regurgitating the digestive fluid from their midgut in a process known as extra-oral digestion (EOD)^4,5^. EOD is complemented by intracellular digestion at the digestive cells. Insects, which are the main prey of spiders, are principally composed of proteins, carbohydrates, and lipids. Thus, spider digestive fluid hydrolases are expected to be capable of catalysing the breakdown of these nutrients. Historically, digestive fluid has been studied for some decades in order to comprehend the enzymes involved in this process. Perret^6^ described the presence of proteolytic activity in venom contaminated with “saliva”, a consequence of digestive fluid extraction by electro stimulation. Mommsen^7–11^ assayed carbohydrases, such as amylase, chitinases, and hexosaminidases as well as peptidases and carboxyl esterases in the digestive juice (term replaced by digestive fluid) of *Tegenaria atrica*. Other studies on digestion in spiders reported the isolation of peptidase from the digestive fluid of *Argiope aurantia*^12–15^ and identification of metallopeptidases in spider digestion and serine peptidase inhibitors in the digestive fluid of this spider^15^. The ultrastructure of the midgut of *Coelotes terrestris* has been described in detail^16^. Joo et al.^17^ isolated a serine peptidase from the whole body of *Nephila clavata* with a pro-thrombin-like specificity. Furthermore, our group described the proteome/transcriptome analysis of spider midgut and its digestive fluid in *Nephilingis cruentata*^18^, elucidating the types and action mechanism of enzymes secreted in EOD and their action role in final intracellular digestion. Walter et al.^19^ corroborated these data through proteomic analyses of the digestive fluid of *Stegodyphus mimosarum* and *Acanthoscurria geniculata*.

Although the importance of digestive enzymes has already been demonstrated in different spider species, many spider venoms also contain different types of enzymes^2,20,21^, such as hyaluronidases, astacins, and serine peptidases. The overlapping of enzymes raises the possibility of contamination of the digestive fluid by spider venom and vice-versa, and the discussion of the importance and balance of enzymes in the digestive fluid and venom for prey digestion is still unclear.

Therefore, spiders without venom would make better models for studying enzymes exclusively involved in prey degradation.

Uloboridae, commonly known as hackled orbweavers, is a spider family composed of 19 genera and 291 species, and is the only spider family that has lost its venom glands secondarily during evolution^22^. Uloboridae spiders are cribellate orb-weavers that exhibit particular prey capture behaviour^3^. Their prey are immobilised by strong and extensive wrapping (up to 7000 wrapping movements), as observed in *Philoponella vicina*^23^, forming a package. Although other spiders only wet the portion close to their chelicerae with the digestive fluid^24^, Uloboridae spiders regurgitate their digestive fluid all over the package surface^25^. Thus, Uloboridae spiders are useful models for understanding the single role of the digestive system in prey degradation.

Based on previous proteomic data of the digestive fluid and the diet of Uloboridae spiders, enzymes such as peptidases, astacin-like metallopeptidases, cysteine peptidases and, carboxypeptidases; carbohydrases, amylase, and chitinases; and lipases were expected to be identified in the digestive tract of Uloboridae spiders. However, some studies have suggested that several enzymes, usually involved in the envenoming process, can also be expressed even in non-venomous animals^26^, highlighting the necessity and importance of comparative studies between the enzymes of the venom and the digestive system. One such enzyme is sphingomyelinase D (SMaseD). SMaseD (EC 3.1.4.41) is a hydrolase which catalyses the sphingomyelin cleavage. Structurally, these enzymes can be divided into two groups: classes I and II. The distinction between class I and II is defined by the presence of a single disulphide bond in class I and two disulphide bonds in class II. Class II is further divided into two subclasses: classes IIa and IIb^27^. Classes IIa and IIb of SMaseD are differentiated based on the dual substitutions at positions 95 (Gly → Asn) and 134 (Pro → Glu) in class IIb^27^. The SMaseD class I acts more specifically to hydrolyse sphingomyelin, class IIa acts on sphingomyelin and more substrates, and Class IIb has non-catalytic or reduced activity against sphingomyelin^27–29^, because of the hydrophilic environment at the entrance to the active site, due to the high polarity of Asn and Glu substitutions^27^.

In the current study, the digestive tract was isolated from *Uloborus* sp., homogenized and, was characterised by electrophoresis separation, enzymatic assays, and proteomic analyses. These data were compared with transcriptomic data from other Uloboridae species and proteomic, transcriptomic, and genomic data from other venomous spider families to understand the enzyme cocktail involved in digestion and envenoming, in order to comprehend the possible shared origin and evolution of enzymatic components in spider digestion and venom.

## Results

### Protein profile of the midgut of Uloborus sp

**Figure 1:**
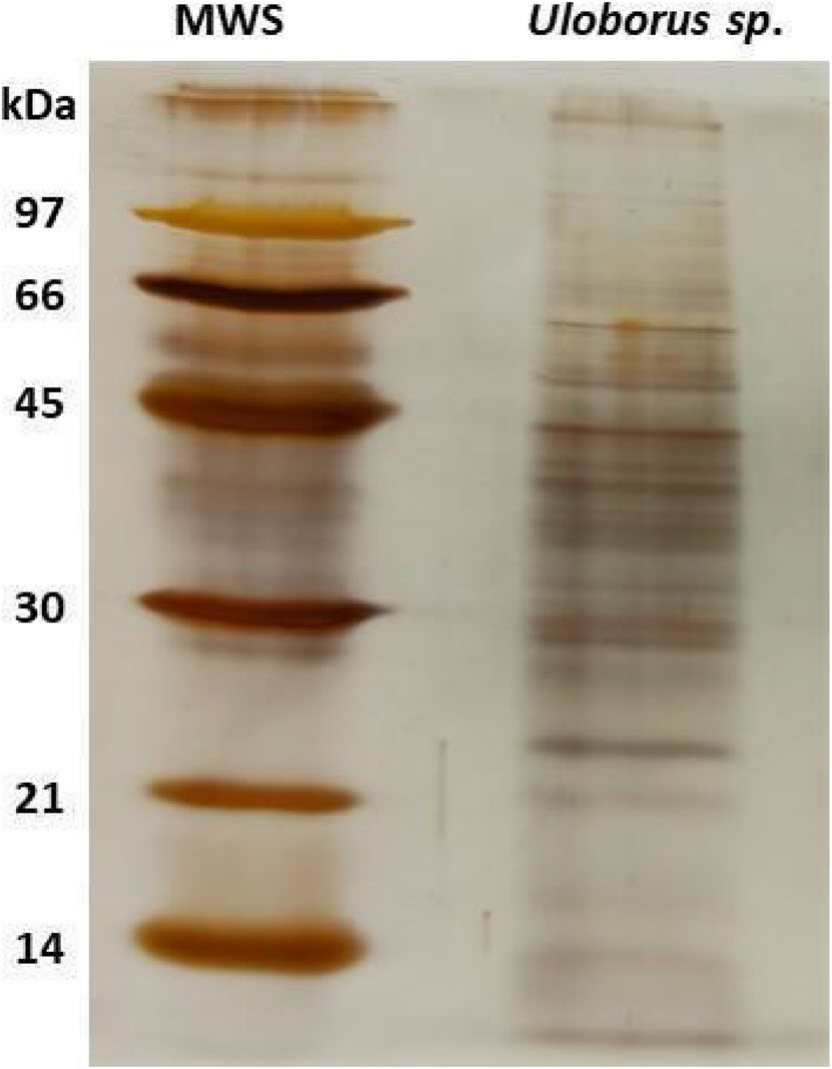
SDS PAGE profile of midgut samples from *Uloborus sp.* About 10 μg of proteins from the midgut of *Uloborus sp.* was submitted to electrophoresis in a 12% polyacrylamide gel at 150 V. MWS—Molecular Weight Standard, 97 kDa—Phosphorylase B, 66 kDa—Albumin, 45 kDa—Albumin from egg, 30 kDa—Carbonic anhydrase, 20.1 kDa—Trypsin inhibitor and 14.4 kDa—Lysozyme. The gel was silver stained according to^76^.

The SDS-PAGE profile in Fig. 1 shows proteins along the gel but is mainly distributed at 66–21 kDa. This protein molecular mass range is compatible with the molecular masses of other digestive enzyme profiles in spiders^18,19^ (Fig. 1).

### Enzymes activities

**Figure 2:**
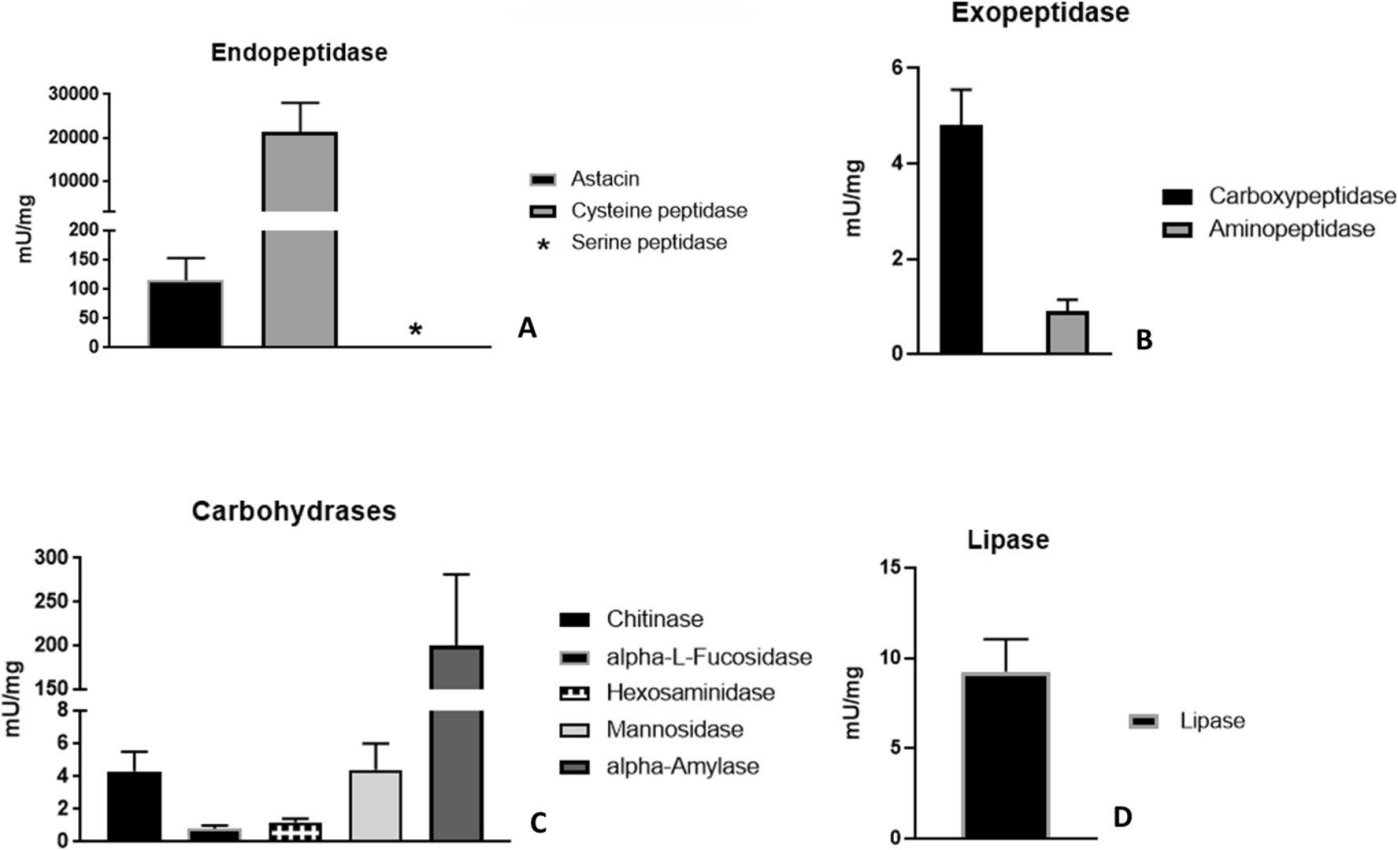
Values of endopeptidases, exopeptidases, carbohydrases and lipase specific activities from the midgut diverticula (MD) of *Uloborus sp.* Enzyme assays were performed as shown in Table 1. N = 5 to endopeptidases (2A); N = 6 to exopeptidases (2B); N = 8 to carbohydrases (2C), except to alpha-amylase (N = 4) and N = 8 to lipase (2D). Colored bars represent specific activity, mean and ± standard deviation of biological replicates for a specific enzyme according to color legend. * Means no activity detected.

According to Fuzita et al.^18^ and Walter et al.^19^, many classes of enzymes have been described in the spider midgut. In this study, 11 different enzymes within three major classes were evaluated: carbohydrases, peptidases, and lipases; the values of enzyme activities are shown in Supplementary Table S1 and Fig. 2.

Cysteine peptidases and astacin showed the major activities of endopeptidases (Fig. 2A), mainly cysteine peptidases (21,400 ± 6700 mU.mg^−1^). No serine endopeptidase activity, such as trypsin or chymotrypsin, was detected despite the different substrates and pH conditions tested, namely: Z-Phe-Arg-MCA, Z-Gly-Gly-Arg-MCA, benzoyl-Arg-pNa, N-succinyl-Ala-Pro-Phe-pNa, and H–D-Phe-Pip-Arg-pNA (thrombin substrate) in a range of pH 3–8 (Table 1) with 10 mM CaCl_2._ Exopeptidases, represented by aminopeptidase and carboxypeptidase, presented activities of 0.9 ± 0.2 mU.mg^−1^ and 4.8 ± 0.7 mU.mg^−1^, respectively, (Fig. 2B).

**Table 1:** Enzymatic assay conditions*.

Enzyme	Buffer	Substrate	Reference
Chitinase	Phosphate-citrate 0.1 M—pH 5.0	MU-N-triacetyl-beta-chitotrioside (1.7 μM)	Baker and Woo (1992)^69^
Hexosaminidase	Phosphate-citrate 0.1 M—pH 5.0	MU-N-acetyl-beta-D-glucosamine (10 μM)	Baker and Woo (1992)^69^
α-Mannosidase	Phosphate-citrate 0.1 M—pH 5.0	MU α-D-mannopyranoside (2 μM)	Baker and Woo (1992)^69^
α-L-Fucosidase	Phosphate-citrate 0.1 M—pH 5.0	MU α-L-fucopyranoside (10 μM)	Baker and Woo (1992)^69^
Cathepsin L	Phosphate-citrate 0.1 M—pH 5.5	Z-Phe-Arg—MCA (10 μM)	Alves et al. (1996)^70^
α-Amylase	Phosphate-citrate 0.1 M—pH 5.5	Starch (from potato) (62 mM)	Noelting and Bernfeld (1948)^71^
Astacin	Tris–HCl 0.05 M—pH 7.2	Casein-FiTC (0.2%)	Twining (1984)^72^
Carboxypeptidase	Tris–HCl 0.25 M—pH 8.0	Z-Gly-Phe (10 mM)	Nicholson and Kim (1975)^73^
Aminopeptidase	Tris—HCl 0.1 M—pH 7.0	L-Leucine-p-nitroanilide (1 mM)	Erlanger et al. (1961)^74^
Lipase	Tris–HCl 0.1 M—pH 8.5	DMPTB (0.22 mM)	Choi et al. (2003)^54^
Serine peptidase	Tris—HCl 0.1 M—pH 3.0—8.0	Z-Phe-Arg—MCA (10 μM)	Alves et al. (1996)^70^
Serine peptidase	Tris—HCl 0.1 M—pH 3.0—8.0	Nα-Benzoyl-L-arginine pNa (1.0 mM)	Erlanger et al. (1961)^74^
Serine peptidase	Tris—HCl 0.1 M—pH 3.0—8.0	Z-Gly-Gly-Arg MCA (10 μM)	Alves et al. (1996)^70^
Serine peptidase	Tris—HCl 0.1 M—pH 3.0—8.0	H–D-Phe-Pip-Arg-pNA (1.0 mM)	Adapted—Erlanger et al. (1961)^74^

*Assay pHs conditions, substrates, references, and their final concentrations in assays, used to measure the enzyme's activities from 11 different enzymes found in *Uloborus sp*. midgut. MU = 4-Methylumbeliferyl; Z = carbobenzoxy; MCA = 7-amido-4-methylcoumarin hydrochloride; DMPTB: 2,3-Dimercapto-1-propanol tributyrate; pNa;p-nitroanilide.

Carbohydrate digestion involves enzymes such as alpha-amylase, chitinase, beta-N-acetyl-glucosaminidase (hexosaminidase), alpha-L–fucosidase, and alpha-mannosidase (Fig. 2, panel C). Alpha-amylase displayed the highest activity (200.2 ± 81.4 mU.mg^−1^) followed by mannosidase (4.4 ± 1.6 mU.mg^−1^), chitinase (4.3 ± 1.2 mU.mg^−1^), hexosaminidase (1.2 ± 0.2 mU.mg^−1^), and α-L-fucosidase (0.8 ± 0.2 mU.mg^−1^). All carbohydrases present an acidic pH optimum (4.5–5.5).

Lipase activity was measured using 2,3-Dimercapto-1-propanol tributyrate (DMPTB) as substrate. This is a generic lipase substrate, which might be hydrolysed by triacylglycerol lipases and allows the hydrolysis by other enzymes such as other esterases, including phospholipases A2 and B. Thus, the measured values of lipase activity (9.2 ± 1.8 mU.mg^−1^) (Fig. 2D) are a sum of these enzymes.

### Proteomic identification

**Figure 3:**
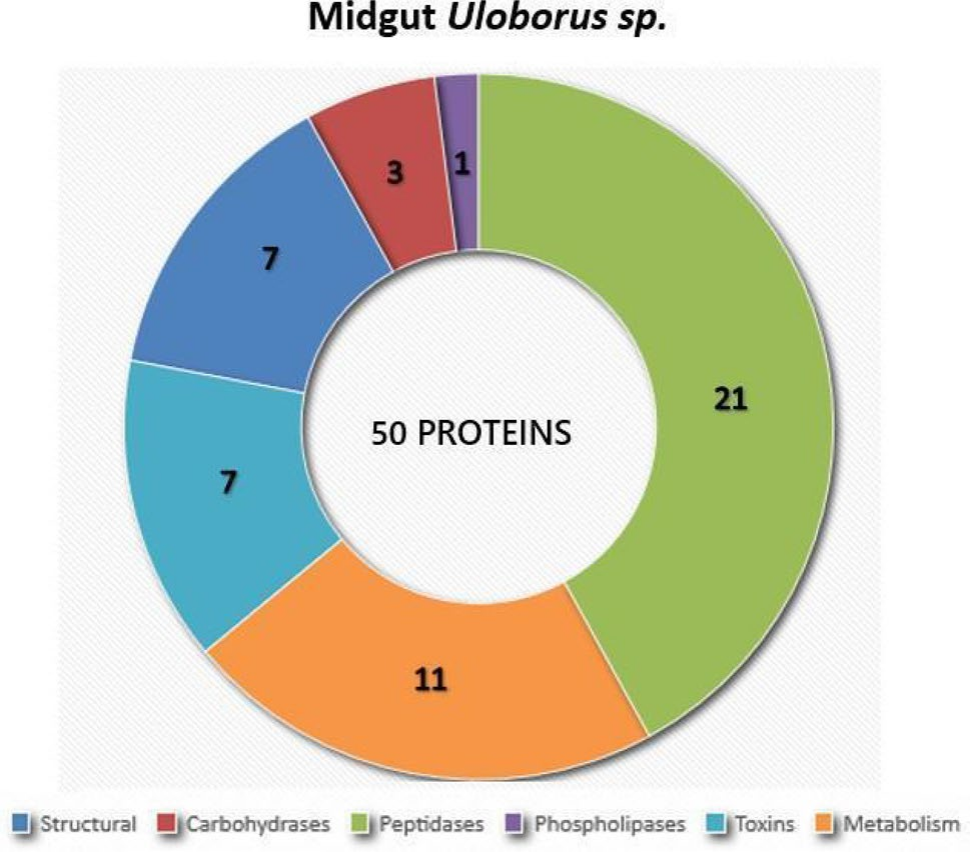
The amount and protein categories by *Uloborus sp*. midgut proteome. Proteins were set apart into six different categories and their amount in *Uloborus sp*. midgut proteome: structural (7), carbohydrases (3), peptidases (21), phospholipases (1), toxins (7) and metabolism (11), summing a total of 50 proteins. Plot was generated in Excel.

In total, 523 tryptic peptides were identified, of which, 50 proteins were matched with different databases. Among the 50 identified proteins (Fig. 3; Supplementary Table S2), 18 were structural proteins such as actin, calmodulin, and alpha-tubulin, or enzymes involved in cell metabolism such as aldehyde dehydrogenase, thioredoxin, and proteasome subunits. The other 33 identified proteins were correlated with proteins already associated with digestion by proteomic analysis of the digestive fluid of *N. cruentata*, *S. mimosarum,* and *A. geniculata*^18,19^, as shown in Supplementary Table S3.

Peptides mainly related to endopeptidases and exopeptidases were most abundant. Cathepsin L, cathepsin O (both cysteine endopeptidases), carboxypeptidase (metallo and serine carboxypeptidases), astacin-like metalloendopeptidases, and serine endopeptidases were the peptidases identified in *Uloborus* sp*.* (Supplementary Table S2). Regarding carbohydrate digestion, 23 peptides related to alpha-mannosidase, chitinase, and alpha-amylase activity were identified. No enzymes belonging to triacylglycerol lipase family were detected. However, an enzyme typical of venom, phospholipase D (or sphingomyelinase, SMaseD), was detected. In addition to phospholipase, U21, U24, and loxtox proteins were also found^30^.

### Sphingomyelinase classification and evolution

**Figure 4:**
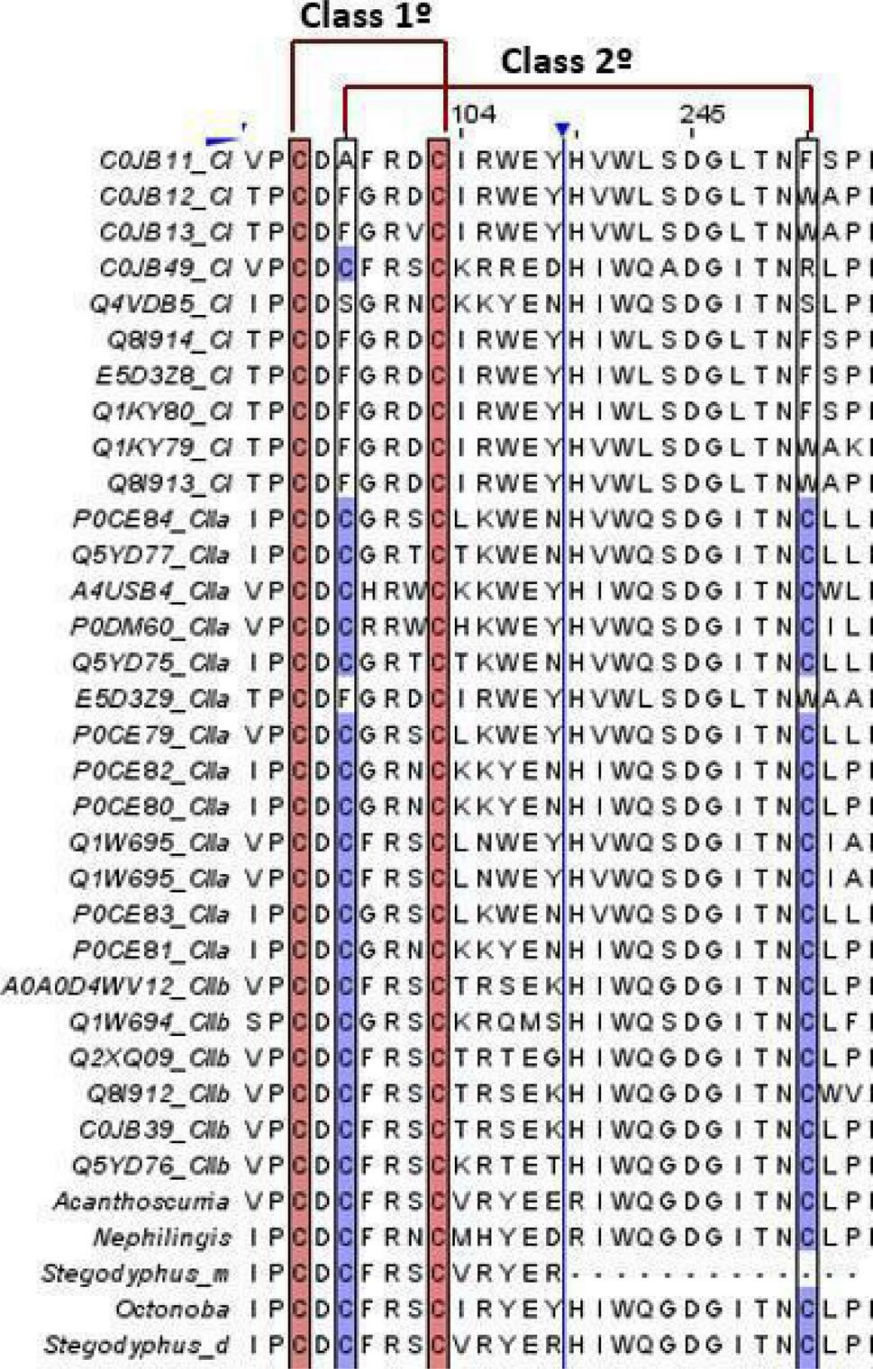
Spider venom and midgut SMaseD multiple sequence alignment and their classification by cysteine conservation. SMaseD of class I, class IIa and class IIb from *L.* venom gland were aligned against SMaseD from abdomen *A. geniculata*, midgut *N. cruentata*, digestive fluid *S. mimosarum,* and whole body *S. dumicola* and *O. yesoensis* in multiple sequence alignment (MUSCLE software), to classify the SMaseD found in the midgut/digestive fluid of spiders. Sequences with only one disulfide bond between Cys97 and Cys103 are classified as “Class I SMaseD” and sequences with an additional disulfide bond between Cys99 and Cys 250 are classified as “Class II SMaseD''. Red box: 1º disulfide bond cysteine conservation; Purple box: 2º disulfide bond cysteine conservation; Blue line/arrow; columns of amino acids positions hidden (95–108 and 240–253). Midgut/digestive fluid SMaseD sequences only conserve the four cysteine residues (Cys97, Cys99, Cys 103, and Cys 250). Alignment was generated in Jalview (https://www.jalview.org)^81^.

**Figure 5:**
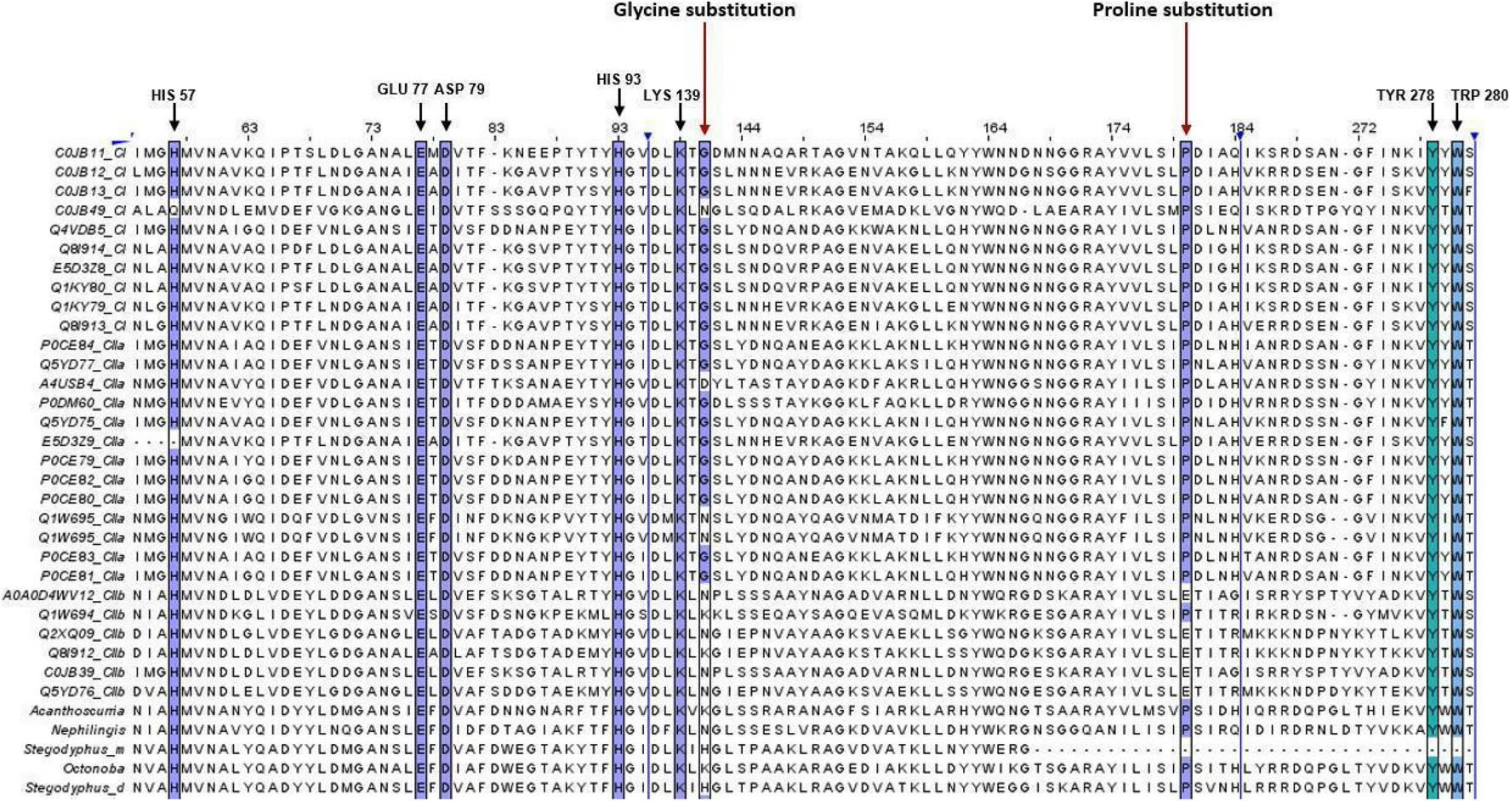
Spider venom and midgut SMaseD multiple sequence alignment, their classification by glycine and proline substitutions, and conservation of residues involved in catalysis. SMaseD of class I, class IIa and class IIb from *L.* venom gland were aligned against SMaseD from abdomen *A. geniculata*, midgut *N. cruentata*, digestive fluid *S. mimosarum,* and whole body *S. dumicola* and *O. yesoensis* in multiple sequence alignment (MUSCLE software) ^80^, to classify the SMaseD found in midgut/digestive fluid of spiders. Most of the class I and class IIa SMaseD conserve the glycine and proline (purple highlighted and red arrowed indicated) residues, and class IIb SMaseD has a substitution in glycine/proline residues. As well, the conservation of histidines (His57 and His93), glutamic acid (Glu77), aspartic acid (Asp79), lysine (Lys139), tyrosine (Tyr278), and tryptophan (Trp280), involved in ion magnesium coordination and substrate recognition. Purple box: conserved histidine, glutamic acid, aspartic acid, lysine, glycine, and proline; Green box: conserved tyrosine; Blue box: conserved tryptophan. Midgut/digestive fluid SMaseD sequences only substitute the glycine residue (Gly141). Alignment was generated in Jalview (https://www.jalview.org^81^.

Proteomic analysis of *Uloborus* sp. digestive system samples showed the presence of SMaseD (Supplementary Table S2). The alignment of spider SMaseD (Figs. 4 and 5) indicate that venom SMaseD belonged to class I and digestive SMase belonged to class IIb.

**Figure 6:**
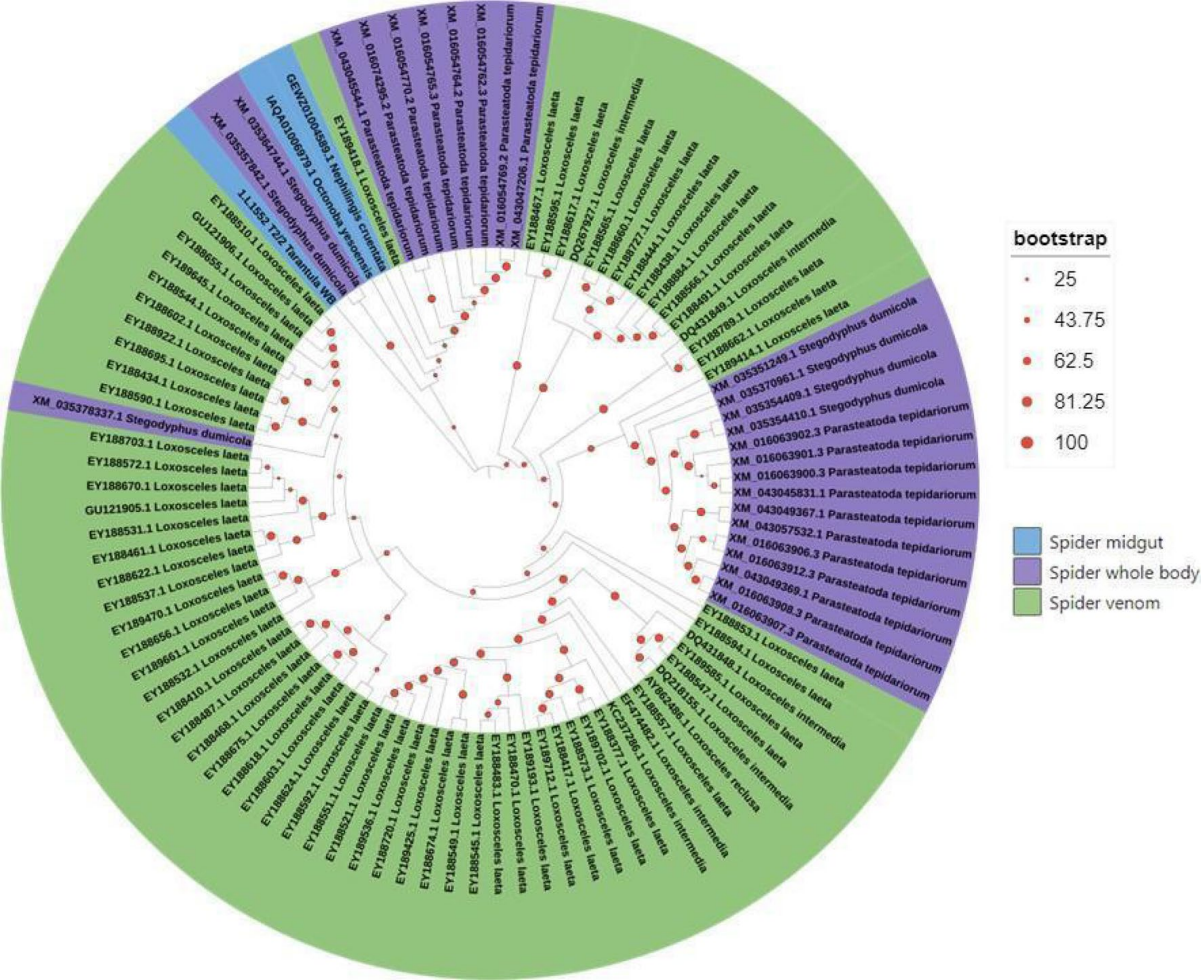
Maximum Likelihood phylogenetic tree from SMaseD sequences. A Maximum Likelihood phylogenetic tree analysis by IQ Tree software^82^ and coloured by ITOL (https://itol.embl.de)^83^, composed of midgut, venom gland sequences from spiders. Bootstrap test of phylogeny was applied with 1000 resampling. All sequences are listed in Supplementary Table S5.

A phylogenetic tree of Chelicerata (Supplementary Fig. S4) has three main groups of SMases represented by distinct Chelicerata species; however, were supported by low bootstrap values. Therefore, a maximum likelihood analysis of SMaseD exclusive from spider SMaseD sequences (midgut and venom) was performed to enhance the statistical bootstrap support (Fig. 6). The tree presents three main groups: (a) a group containing the proteome confirmed digestive SMaseD, classified as SMaseD IIb; (b) a group containing other spider venom and some *Loxosceles laeta* and *L. intermedia* SMaseD II; and (c) a group containing majorly *L. laeta*, *L. intermedia* and *L. reclusa* venom SMaseD class I (Fig. 6).

## Discussion

Due to lack of venom, Uloboridae spiders have developed unique strategies for prey capture, prey handling, and digestion. These strategies include behavioural changes such as resting and feeding positions. Several Uloboridae species rest on their webs in a cryptic position to hide their anterior legs for protection against predators^31^. However, members of the genus *Uloborus*, for example, change these postures during feeding. Esquivel et al.^24^, showed that the digestive fluid of these spiders damages their legs, indicating that the leg position changing behaviour during feeding is to avoid leg damage, suggesting that digestive fluid is rich in hydrolytic enzymes. However, to date, specific digestive enzymes involved in prey liquefaction are not known in this group of spiders. Insects, the main Uloborid prey, have a higher percentage of proteins than carbohydrates and lipids^32^ in their body composition. Therefore, high activities or many digestive peptidases are expected in Uloboridae because total protein ingestion is the sum of protein content in prey and protein present in silk consumption from the wrapping envelope^33^.

Biochemical assays of soluble fraction of the digestive tract of *Uloborus* sp*.* revealed endopeptidase and exopeptidase activity (Supplementary Table S1 and Fig. 2A,B). Regarding exopeptidase activities, *Uloborus* sp. presented similar enzyme activities and peptides as other spider species, corroborating the data that carboxypeptidases are the predominant exopeptidase enzymes involved in protein degradation. The identified carboxypeptidases belonged to the serine carboxypeptidase (EC 3.4.16.6) and metallocarboxypeptidases (EC 3.4.17.17; EC 3.4.17.24) families. Similar results were obtained for previously characterised spiders, indicating that exopeptidases are important components secreted during the first phase of protein digestion.

The endopeptidases, astacin and cysteine peptidases, were identified by enzyme activity and peptide identification by mass spectrometry, as expected based on the literature data of spiders’ digestive fluid. The involvement of astacin in Uloboridae digestion was expected because of its specificity and the composition of the web used for prey immobilisation^34,35^. Astacin preferentially hydrolyses peptide bonds formed by Ala, Gly, and Ser residues at P1 and P1’^36^.

In contrast, the Uloboridae catching web comprises different types of spidroins, including aciniform spidroins (AcSp). The AcSp rich in alanine (Ala), glycine (Gly), and serine (Ser) forms aciniform spidroins type 1 (AcSp1)^37,38^, which is suitable for astacin hydrolysis. The importance of the spider digestive fluid has been demonstrated in the degradation of the thinner type-A aciniform spidroin lines during Uloboridae feeding.

However, contrary to the expected, the specific activity of Uloborus sp. astacin was lower than that of other spiders astacins. Proteomes from *N. cruentata* digestive tract and also from the digestive fluids from *N. cruentata*, *S. mimosarum,* and *A. geniculata* show a higher proportion of astacins than other endopeptidases, mainly, by an abundant presence of astacins isoforms^18,19^, respectively 27, 34 and 11 astacins were identified in the digestive fluids of spiders. However, *Uloborus* sp. midgut proteomic data revealed only two sequences of metallopeptidases. The lower astacin content identified in *Uloborus* sp. might be related to the distinct mass spectrometry methods applied to sample analysis. However, proteomic data from other spider samples using the same methodology allowed the identification of more astacin peptides than those identified in *Uloborus* sp. Another limitation in the identification of astacin peptides may be related to the absence of *Uloborus* sp*.* transcriptome data. The proteomic analysis was done using a database composed mainly of *Araneus ventricosus* and *S. mimosarum* sequences which are the best databases available of spiders and from the transcriptome of the Uloboridae spider *Octonoba yesoensis*^39^. The transcriptome of *O. yesoensis* has 50 distinct transcripts of astacins, which is a compatible number of astacins in spiders^18,19^, possibly indicating that the low activity and peptides of *Uloborus* sp. astacin could still be related to other factors such as astacin specificity/assay substrate and feeding conditions.

Walter et al.^19^ suggested that the difference between astacin numbers could be related to web degradation and the evolutionary scale of different spiders. To understand this, other specific transcriptome and proteome data are required.

Although astacins and cysteine peptidases are the main peptidases in spiders, another family of endopeptidases, namely serine peptidase, might be involved in protein degradation. Many peptides from the S1 (serine peptidase) family have been identified by proteomic analysis in all spiders in which digestive enzymes were studied^18,19,40^. However, identification of serine peptidase activity was not possible even with different substrates and assay conditions. The only measured serine peptidase activity was observed in *Trichonephila clavipes*^17^, indicating enzyme specificity similar to that of thrombin hydrolysis. Serine peptidases are also present in the EOD enzyme cocktail, although the expressed trypsins in the midgut of spiders seem to be different from classical trypsins, such as bovine and human trypsins, owing to the presence of accessory domains^40^. Our research group identified accessory domains analysing the data obtained from the midgut proteome and transcriptome of *N. cruentata*^18^, digestive fluid of *A. geniculata,* and *S. mimosarum*^19^, and the whole body transcriptome of *O. yesoensis*^39^.

Acidic protein degradation catalysed by cysteine peptidase is also important, as previously reported^18,19,41^ and is involved in both, the extracellular digestion during prey liquefaction, and intracellular digestion. Our results also demonstrate the participation of cathepsin L-like enzymes in the digestive process of *Uloborus* sp*.* (biochemically and by proteomic analysis) and from the transcriptome from *O. yesoensis*. Thus, the presence of acidic peptidases is mandatory in spider digestion, even in the digestive fluid and midgut digestive cells.

As described by^18,41^, enzymes that are mainly involved in intracellular digestion, such as cysteine peptidases, alpha-L-fucosidase, and alpha-mannosidase, perform a more acid hydrolysis, and secreted enzymes, such as chitinases, astacins, alpha-amylase, aminopeptidases, and carboxypeptidases conduct a less acidic EOD. The pattern of enzymes pH optimum, which correlates with enzymes location/phase of digestion, is the same in *Uloborus* sp. as that described for other spiders^18,19,41^.

### Carbohydrates digestion

The main enzymes involved in carbohydrate digestion in spiders are chitinases and hexosaminidases (β-N-acetylglucosaminidase), which act synergistically to catalyse the cleavage of chitin polymers, and amylase. Chitinase and β-N-acetylglucosaminidase were firstly isolated from *Cuppienius salei*^11^ and their activities are related to insect carcass degradation, while promoting the accessibility of the venom and the digestive fluid to prey tissues, as well as a defensive role against microorganisms.

Amylase catalyses the cleavage of the α-1,4-glycosidic bonds in starch and glycogen. In insects, glycogen is abundantly stored in the fat body^42^. However, starch is also an available substrate for spider amylase. Starch can be present in the pollen grain, adhered to the spider’s web, and ingested during web recycling^5^; it also could be attached with a prey that is a pollinator, or even in the midgut content at digestive tract of herbivorous insects ingested as prey^43,44^. Furthermore, actively feeding on pollen is important for orb-weaver spider nourishment, particularly for juvenile orb-weaver’s spiders^45^. *Uloborus* sp*.* presented both alpha amylase activity, identified biochemically, and alpha amylase peptides in the proteomic analysis. Measurements of enzymatic activity using glycogen and starch as substrates indicated that *Uloborus* amylase evenly hydrolysed both substrates.

Proteomic and biochemical data also identified enzymes involved in the removal of carbohydrates from glycoconjugates, including alpha-L-fucosidase and alpha-mannosidase which are related to the intracellular digestive process in *Uloborus* sp. The first characterisation of a spider alpha-L-fucosidase was obtained from the midgut of *N. cruentata*^46^. In insects, the N-glycosylation pathways involve a high mannosylation and two fucosylations (Man3GlcNAc(± α3/6Fuc) GlcNAc) of glycoproteins, forming a structure known as paucimannosidic glycans^47^. Thus, alpha-mannosidase and alpha-L-fucosidase of spiders participate in the removal of these glycan residues during prey digestion. Furthermore, diverse glycans repertoires participate in host–parasite relationship^48^, in glycoproteins and glycolipids so-called ‘host-like’ glycans^49^. In this manner, alpha-mannosidase and alpha-L-fucosidase could also play defensive roles^50^.

### Lipid digestion

Different types of triacylglycerol, diacylglycerol, and monoacylglycerol lipases, and phospholipases are involved in lipid and phospholipids hydrolysis.

Lipid storage (lipid droplets) in the midgut of spiders is essential for nourishment, during extensive periods of starvation^51^, and for delivering lipid pathways to other tissues^52^. Lipids are one of the major macronutrients in insect^53^, thus, lipases are crucial for lipid digestion in spiders.

The midgut proteome of *Uloborus* sp. identified only phospholipases, such as phospholipases C and D. Lipase activity was measured using DMPTB. This activity may be related to hydrolysis by non-specific lipases^54^. Therefore, triacylglycerol, diacylglycerol, and monoacylglycerol lipases could hydrolyse DMPTB, endorsing the presence of lipases in *Uloborus* sp. midgut; as well as phospholipases C release diacylglycerol during phospholipids hydrolysis, making it a suitable product for lipases.

The midgut of *Uloborus* sp., used as a source of digestive enzymes in the present study, has limited information on the feeding conditions of the individuals studied, but they were at least 72 h fasting. As observed in *Pardosa prativaga*, a higher metabolism rate was observed in the first 3 h of digestion^55^, and in *N. cruentata*, there are more lipases in animals under fasting conditions^18^. Thus, feeding conditions could be the reason that we did not identify lipases in *Uloborus* sp. midgut proteome. In addition, as the midgut diverticula are one of the tissues with higher volumes in a spider, the whole-body transcriptome of *O. yesoensis*, an Uloboridae spider, revealed 21 lipase transcripts, some of which might belong to the midgut diverticula.

### SMaseD phylogenetics

Some peptides identified in the proteomic analysis of *Uloborus* midgut are orthologues of peptides identified in the venom gland of other non-Uloboridae spiders, corroborating previous data reported in the literature that some peptides with toxin function are expressed in tissues distinct from the venom gland. Previously, the identification of some proteins homologous to venom toxins has been attributed to digestive fluid contamination with venom. Foradori et al.^15^ were the first to isolate several peptidase inhibitors similar to those identified in spider venom in the digestive fluid of *A. aurantia*. The transcriptome of *N. cruentata* (GenBank: GEWZ00000000.1)^18^ allowed the identification of toxin and enzyme orthologues transcripts of spider midgut to those found in venoms.

These toxin-like peptides have already been identified in other non-venomous Arachnida, such as mites and ticks^26^. Similar to peptide toxins, enzymes involved in the envenomation process, which are exclusive to venom, are also shared between the venom gland and other organs and have been described in mites and ticks. SMaseD is an example of such enzyme.

Although SMaseD in Arachnida is a group of orthologous genes, it can have distinct disulphide binding patterns and activities. SMaseD is expressed in the salivary glands of ticks and modulates the host immune system, facilitating pathogen transmission^26^. Some tick genera, such as *Rhipicephalus* and *Amblyomma*^56^, cause inflammation and necrosis in the host. However, recombinant SMaseD from *Ixodes scapularis* did not show necrotic effects. Differences in SMaseD catalytic efficiency and specificity have also been observed between Sicaridae species^57^.

Our data on proteomic analysis of the digestive tract of *Uloborus* sp. and the analysis of the transcriptome of *O. yesoensis* suggested the presence of SMaseD in Uloboridae spiders. The identification of toxins including SMaseD, in the midgut of *Uloborus* sp. is a complete proof of the involvement of this enzyme in digestion with potential insecticidal activity, facilitating prey paralysis and capture even in a non-venomous spider.

Analysis of the midgut transcriptome and proteome from *N. cruentata*, of the opisthosoma (abdomen) of *A. geniculata*^19^, and the midgut proteome of *Loxosceles gaucho*^58^ also identified SMaseD involved in the digestive tract. The analysis of *S. mimosarum* digestive fluid proteome identified peptides corresponding to SMaseD, but in low concentration. In contrast, analysis of the proteome of the digestive fluid from *N. cruentata* and *A. geniculata* did not detect SMaseD peptides. These data suggest the involvement of SMaseD mainly in the intracellular phase of digestion. This is in accordance with the SMase identified in tick genomes, which are predicted to be acidic and neutral SMases, localised in the lysosome.

SMase multiple alignment allowed the maximum likelihood algorithm to expand the history of the SMaseD gene in some Chelicerata species (Supplementary Fig. S4). Orthologues of SMaseD genes were identified even in *Limulus* transcriptome data (Supplementary Table S5), a basal group of the Chelicerata subphylum, suggesting that the association of SMaseD with digestion is older than its relation to envenomation in spiders. Another aspect observed in this analysis is an unusual distribution of ticks and mites SMaseD, although both groups possess class IIb SMaseD, mite SMaseD has sequences represented in the three groups distinguished in the tree; however, most sequences are more similar to SMasesD from spiders midgut than from ticks SMaseD. Usually, regarding other digestive enzymes, mites and ticks present sequences with high identity values and are frequently grouped together^18,41^ indicating a distinct selective pressure to the evolution of SMaseD.

Although this tree is informative, it has low bootstrap values support. In order to enhance the statistical bootstrap support, and evidence the grouping of the distinct classes of SMase in spiders, an exclusive spider maximum likelihood tree was performed.

The exclusive spider analysis and sequence alignment indicated that the midgut SMaseD sequences belong to the class II SMaseD subfamily, conserving the two disulphide bridges (Fig. 4) between Cys97 and Cys103 (corresponding to Cys51 and Cys57, according to PDB entry)^27,59^ and an additional disulphide bridge between Cys99 and Cys250 (corresponding to Cys53 and Cys201, according to PDB entry)^27,59^. Moreover, alignment analysis suggested that digestive SMaseD belongs to class IIb, due to substitution at Gly141 (corresponding to Gly95, according to PDB entry)^27^ by an asparagine, lysine, or histidine residue (Fig. 5); and conservation of proline residue Pro180 (corresponding to Pro134, according to PDB entry)^27^. Furthermore, midgut SMaseD conserves the histidine, glutamic acid, and aspartic acid residues: His57, His93, Glu77, and Asp79 (Fig. 5), according to LiRecDT1^60^, involved in the catalytic process and coordination of magnesium ion at the active site^28^, as well as, conserves lysine, tyrosine, and tryptophan residues: Lys139, Tyr278, and Trp280 (Fig. 5), according to LiRecDT1, involved in substrate recognition and stabilization in the cleft of the active site^28^.

Previous analyses^27,59^, comparing the relationship and adaptive evolution of SMaseD to glycerophosphodiester phosphodiesterase were so far made, exhibiting a positive selection on spider venom SMaseD, and an increase of necrotic activity.

Although SMaseD has been widely described for their toxicity in mammals, the main prey of these spiders, even of the family Sicaridae, are other arthropods. *Loxosceles laeta*, *Loxosceles intermedia* and *Loxosceles arizonica* for example have, as their main prey, other spiders, ants and scorpions. On the other hand, little is known about lipidic constituents in arthropods. In Diptera the main complex lipids are ceramide phosphoethanolamines. However, in other groups, such as hemipterans the main lipids are sphingomyelin^61^. Diets composed of prey rich in sphingomyelin constitute an important selective pressure on more effective SMaseD, culminating in the selection of insecticide molecules.

Another aspect that should be emphasised is the common enzyme between the salivary glands of ticks and the venom gland of spiders. One of the hypotheses of their appearance in the venom gland in spiders is that they originate from the salivary glands^2,62^, concomitantly with the gene duplication events in the Arachnida and Chelicerata groups^63–66^. Our results suggest that the spider venom gland origin is closely related to the salivary gland in ticks and is derived from a gland associated with digestion because of the presence of class IIb SMaseD and other shared components between these tissues.

## Conclusion

Here, we summarise for the first time the profile of the midgut enzymes of the non-venomous spider *Uloborus* sp*.*, using proteomic and enzymological approaches. The results indicated that *Uloborus* sp. shares the main digestive enzymes, such as metallopeptidases and cysteine peptidases, carboxypeptidases, chitinases and amylases, with the families of venomous spiders. In addition to typical digestive enzymes, the midgut of Uloboridae also contains enzymes such as SMaseD and other toxin peptides usually found in venom glands, suggesting an insecticide activity of digestive fluid in Uloboridae and indicating that the origin of spider toxins during evolution is possibly the digestive system.

## Material and methods

### Animals and sample preparation

Adult females of *Uloborus sp.* were collected in Fazenda Nova Monte Carmelo, located between municipalities of Araguari, Romaria, Nova Ponte, Estrela do Sul and Monte Carmelo, in Minas Gerais, Brazil (18°49′27″S, 47°51′47″W). The area is an *Eucalyptus* crop interspersed with native Cerrado vegetation (i.e. Brazilian.

**Figure 7:**
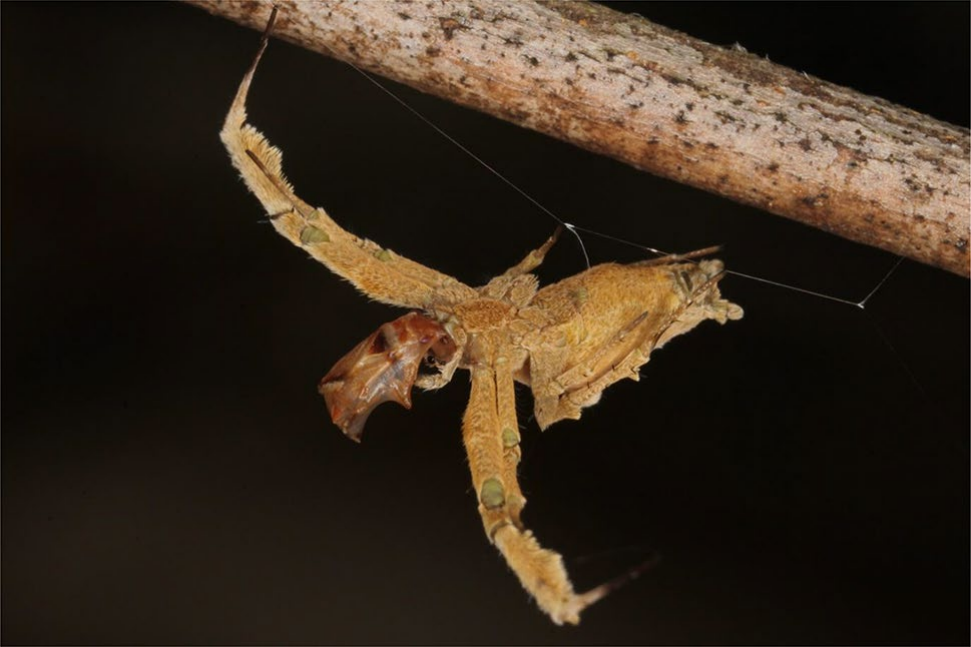
Female of *Uloborus sp*. feeding on a prey item captured in the field (picture from Marcelo de Oliveira Gonzaga).

Savanna). As all of the Brazilian *Uloborus* species have been described based only on color patterns, species identification still requires a taxonomic revision of South American species. The species object of this study in Fig. 7 is the same previously studied by Nascimento^67^ and voucher specimens were deposited in the collection of Universidade Federal de Minas Gerais (Reference number: 16685).

Sampled individuals were immediately sent to Instituto Butantan (São Paulo, SP) and were kept in 15 mL tubes, at room temperature, for at least 72 h of fasting. The spiders were then anesthetized in CO_2_ for about 10–15 min and subsequently dissected, using a stereomicroscope and isotonic saline solution (NaCl 300 mM)^5^ for the isolation of the midgut, silk gland, egg sac, and carcass. The different tissues were collected and stored at −20ºC.

Midguts were individually homogenized with a Potter–Elvehjem to fragment the internal membrane of enterocytes and release content, using cold Ultrapure Water (Milli-Q). Thereafter, the homogenate was centrifuged at 16,000 × g for 30 min at 4ºC. The soluble fraction was used as an enzyme source, and it was stored at −20ºC.

### Protein determination and enzymatic assays

The protein content from *Uloborus sp* midgut samples was estimated by the bicinchoninic acid method^68^ using albumin from the chicken egg as standard protein. Enzymatic assays were performed at 30 °C using a series of chromogenic and fluorescent substrates. Product reaction was measured using a spectrophotometer (SpectraMax M2—Molecular Devices) and spectrofluorometer (SpectraMax Gemini XPS—Molecular Devices), through at least four different intervals of time to ensure enzyme initial velocities. Blanks of the enzyme (enzyme plus buffer at the same assay condition) and the substrate (substrate plus enzyme solvent) were used as negative controls. Enzymes, buffers, substrates, and their final concentrations at the well plate are listed below (Table 1). At least, 5 different biological samples were used to each enzyme assay.

### Sodium dodecyl sulfate–polyacrylamide gel electrophoresis (SDS-PAGE)

SDS-PAGE was performed with one individualized midgut homogenized in 500 μl of ultra-filtered cold water. The homogenate sample was submitted to VivaSpin 20 centrifugal concentrator MWCO 10 kDa using ultrafiltered water as diluent was concentrated 500 μl of homogenate sample, which corresponds to, was resuspended in 20 μl of sample buffer (approximately, a total of 10 μg). Samples from *Uloborus* sp*.* midgut (approximately 10 μg of protein) were submitted to VivaSpin 20 centrifugal concentrator MWCO 10 kDa using ultrafiltered water as diluent. After that, samples were concentrated in vacuum centrifugation (speed vac), and resuspended (total of 10 μg of protein) in 20 μl of sample buffer, containing: 60 mM Tris–HCl buffer (pH 6.8), 2.5% SDS, 0.36 mM b-mercaptoethanol, 10% (v/v) glycerol, and 0.005% (w/v) bromophenol blue, thereafter, the sample was heated at 100 ºC for 5 min. Samples were applied onto a 12% polyacrylamide gel according to^75^. The electrophoresis was performed at 150 V, and the gel was silver stained^76^.

### Bottom-up proteomics

An aliquot of *Uloborus* sp*.* midgut was concentrated in vacuum centrifugation (speed vac), to a total of 10 μg of protein. Afterward, the procedure of digestion in solution was: 20 μl of 8 M Urea, 10 min at 22 °C, 0.5 μl of 1 M dithiothreitol (DTT) for 30 min and 30 °C, 5 μl of 200 mM iodoacetamide (IAA) in the dark for 30 min at 22 °C, 90 μl of digestion buffer [50 mM NH_4_HCO_3_ + 10% acetonitrile (ACN)] to dilute urea concentration and addition of trypsin to protein ratio of 1:100. The digestion was incubated for 18 h at 30 °C and stopped by the addition of 0.1% formic acid (FA). After that, ACN was removed by vacuum centrifugation.

The sample concentrated by vacuum centrifugation was resuspended in 50 μl of acidic solution (0.1% FA) to mass spectrometry procedure. Tandem mass spectrometry analysis of tryptic peptides was performed by an LC–MS/MS Q-TOF (Shimadzu), samples (50 µL aliquot) were loaded into a C18 column (Kinetex C18, 5 μm; 50 × 2.1 mm) and peptides eluted by binary gradient of 5% to 40%, solvent A—Water:FA (999: 1) and solvent B—ACN:Water:FA (900: 99: 1), at a constant flow of 0.2 mL.min^-1^ for 40 min.

Raw LCD, LCMSolution—Shimadzu, data were converted into MGF by the LCMSolution tool and then loaded into Peaks Studio V7.0 (BSI, Canada). Data were processed according to the following parameters: MS and MS/MS error mass were: 0.1 Da; methionine oxidation and carbamidomethylation as variable and fixed modification, respectively; trypsin as cleaving enzyme; maximum missed cleavages (3), maximum variable PTMs per peptide (3) and non-specific cleavage (both); only proteins with score ≥ 40 and containing at least 1 unique peptide were considered in this study.

### Bioinformatic analysis/databases

Different databases from spiders employed these searches, such as *N. cruentata* midgut proteome^18^, *S. mimosarum,* and *A. geniculata* genome^77^, and their digestive fluid proteome^19^. Furthermore, we employed Trans Decoder^78^ (Galaxy Australia), finding coding regions software, to the raw database from *O. yesoensis* whole body transcriptome (GenBank: IAQA00000000.1), afterwards, the translation and annotation of the enzyme's nucleotide sequences were obtained by blastp, using *A. ventricosus* and *S. mimosarum* amino acid enzyme sequences as queries.

Protein sequences of SMaseD Class I, IIa, and IIb were obtained at: Uniprot reviewed database (venom gland *Loxosceles* genera);^18^ SMaseD from the midgut of *N. cruentata*;^39^, from the whole body of *O. yesoensis*;^77^ transcriptomic data from *S. mimosarum* and *A. geniculata* corroborated by proteomic data from the spider´s digestive fluid and/or abdomen^19^, and submitted to multiple sequence alignment analysis and classification of midgut SMaseD sequences. SMaseD sequences from *O. yesoensis* were acquired by Trans Decoder^78^ (Galaxy Australia) annotation of the transcriptome (GenBank: IAQA00000000.1), filtered SMaseD nucleotide sequences using *N. cruentata* as Blastn query (NCBI), and translated to amino acid sequence by Translate Tool (Expasy)^79^. Protein alignment of SMaseD sequences was built by MUSCLE software^80^ and analyzed in Jalview (2.11.2.2 version) software^81^, in order to compare the cysteine residues conservation and pattern and glycine/proline residue substitutions at specific positions to enzyme classification.

For the construction of an SMaseD phylogenetic tree of Arachnida and Chelicerata species we collected nucleotide SMaseD sequences from midgut *Tityus serrulatus*, *Ixodes ricinus*, *Rhipicephalus microplus,* and *Amblyomma aureolatum*, from the salivary gland of *Ixodes scapularis*, from whole body *Dermacentor silvarum*, *Rhipicephalus sanguineus*, *Tetranychus urticae*, *Metaseiulus occidentalis* and *Limulus polyphemus*, venom gland *Hemiscorpius lepturus*; sequences were collected by keywords (Sphingomyelinase D, Phospholipase D and Dermonecrotic toxin) search in Nucleotide Advanced Search Builder (NCBI), filtered to Arachnida group and only mRNA and EST sequences were selected. Furthermore, midgut sequences were also added to the construction at the phylogenetic tree. The sequences and access codes are listed in Supplementary Table S5.

The Nucleotide sequence alignment was performed by MUSCLE software^80^. The phylogenetic tree was constructed using by IQ Tree (Galaxy Version 2.1.2 + galaxy2) software^82^ by a “Maximum Likelihood Tree”, with a bootstrap method as a test of phylogeny with 1000 bootstrap replications. The trees were coloured using iTol (Interactive tree of life) v6 software^83^.

## Supplementary Information

Supplementary Information.

## Data Availability

The datasets generated and/or analyzed during the current study are available in: 1- Proteomic data: the PRIDE repository, Data are available via ProteomeXchange with identifier PXD037345 Project https://doi.org/10.6019/PXD037345. Reviewer account details Username: reviewer_pxd037345@ebi.ac.uk Password: OafQcthc. (https://www.ebi.ac.uk/pride/login). 2- The translated sequences from *Octonoba yesoensis* ^39^—https://repositorio.butantan.gov.br/handle/butantan/4540.
