# Supplementary material for: Digestive enzymes and sphingomyelinase D in spiders without venom (Uloboridae)
Source: Sci Rep. 2023 Feb 15;13:2661. doi: 10.1038/s41598-023-29828-x (PMC9932164; doi:10.1038/s41598-023-29828-x)
Supplement: Supplementary file 1 — Supplementary Information. [file 41598_2023_29828_MOESM1_ESM.docx]

**Supplementary Table S1** Values of absolute and specific activities from midgut enzymes of *Uloborus sp*.•

|  | | |
| --- | --- | --- |
| **ENZYMES** | **AA** | **SA** |
| **Chitinase** | **4.3 ± 1.5** | **4.3 ± 1.2** |
| **α-L-Fucosidase** | **0.6 ± 0.2** | **0.8 ± 0.2** |
| **β-Hexosaminidase** | **0.7 ± 0.1** | **1.2 ± 0.2** |
| **α-Mannosidase** | **0.3 ± 0.01** | **4.4 ± 1.6** |
| **α-Amylase** | **155.8 ± 63.8** | **200.2 ± 81.4** |
| ****Cysteine peptidase** | **12.7 ± 3.8** | **21.4 ± 6.7** |
| ***Astacin** | **0.8 ± 0.2** | **114.6 ± 38.7** |
| **Carboxypeptidase** | **3.0 ± 0.5** | **4.8 ± 0.7** |
| **Aminopeptidase** | **0.5 ± 0.1** | **0.9 ± 0.2** |
| **Lipase** | **6.4 ± 1.1** | **9.3 ± 1.8** |
| **Serine Peptidase** | **0** | **0** |

•The table represents the values of absolute and specific activities from midgut *Uloborus sp*. enzymes and their respective standard mean deviations. AA: Absolute activity (mU.animal^-1^ SA: specific activity (mU.mg^-1^); *: AA- (Abs/min). animal^-^¹; **: AA - U.animal^-^¹ and SA - U.mg-¹. At least N=5 biological replicates to each enzyme.

**Supplementary Table S2 -** Identified proteins in Uloborus sp. midgut proteome

| **Specie** | ***Acession*** | ***-10lgP*** | ***Coverage (%)*** | ***Peptides*** | ***Uniques*** | ***Avg. Mass (Da)*** | ***Description*** |
| --- | --- | --- | --- | --- | --- | --- | --- |
| ***Stegodyphus mimosarum*** | ***A0A087TZB3*** | ***101.86*** | ***17*** | ***4*** | ***4*** | ***29279*** | ***14-3-3 protein epsilon (Fragment)*** |
| ***Stegodyphus mimosarum*** | ***A0A087UI49*** | ***185.44*** | ***19*** | ***7*** | ***7*** | ***41289*** | ***Actin clone 403 (Fragment)*** |
| ***Carios mimon*** | ***A0A147BB59*** | ***57.69*** | ***5*** | ***2*** | ***0*** | ***53363*** | ***Aldehyde mitochondrial (Fragment)*** |
| ***Ixodes scapularis*** | ***B7PNG4*** | ***69.58*** | ***7*** | ***1*** | ***1*** | ***30772*** | ***Alpha tubulin putative*** |
| ***Stegodyphus mimosarum*** | ***A0A087SXW7*** | ***71.42*** | ***15*** | ***15*** | ***15*** | ***75839*** | ***alpha-1 2-Mannosidase (Fragment)*** |
| ***Parasteatoda tepidariorum*** | ***A0A2L2XY79*** | ***48.80*** | ***3*** | ***2*** | ***1*** | ***57114*** | ***Alpha-amylase (Fragment)*** |
| ***Stegodyphus mimosarum*** | ***A0A087TSS0*** | ***75.45*** | ***12*** | ***13*** | ***13*** | ***149950*** | ***Angiotensin-converting enzyme (Fragment)*** |
| ***Ixodes ricinus*** | ***A0A0K8RMV8*** | ***84.42*** | ***15*** | ***2*** | ***2*** | ***17503*** | ***Annexin (Fragment)*** |
| ***Tetranychus truncatus*** | ***A0A3G5AP06*** | ***129.93*** | ***18*** | ***7*** | ***7*** | ***41808*** | ***Beta-actin*** |
| ***Stegodyphus mimosarum*** | ***A0A087UZS7*** | ***96.47*** | ***16*** | ***2*** | ***2*** | ***16811*** | ***Calmodulin (Fragment)*** |
| ***Tropilaelaps mercedesae*** | ***A0A1V9XF89*** | ***62.46*** | ***6*** | ***1*** | ***1*** | ***28725*** | ***Cam protein*** |
| ***Stegodyphus mimosarum*** | ***A0A087T9Q9*** | ***68.97*** | ***9*** | ***5*** | ***5*** | ***54424*** | ***Carboxypeptidase (Fragment)*** |
| ***Stegodyphus mimosarum*** | ***A0A087U2P7*** | ***74.93*** | ***3*** | ***8*** | ***8*** | ***190673*** | ***Carboxypeptidase D (Fragment)*** |
| ***Stegodyphus mimosarum*** | ***A0A087UTY2*** | ***62.03*** | ***9*** | ***4*** | ***3*** | ***34793*** | ***Cathepsin L (Fragment)*** |
| ***Stegodyphus mimosarum*** | ***A0A087V0D1*** | ***43.77*** | ***5*** | ***3*** | ***3*** | ***39054*** | ***Cathepsin O (Fragment)*** |
| ***Stegodyphus mimosarum*** | ***A0A087U201*** | ***95.50*** | ***15*** | ***16*** | ***15*** | ***99256*** | ***Coagulation factor X (Fragment)*** |
| ***Stegodyphus mimosarum*** | ***A0A087U2C0*** | ***75.80*** | ***16*** | ***12*** | ***12*** | ***58124*** | ***Complement factor B (Fragment)*** |
| ***Stegodyphus mimosarum*** | ***A0A087TIJ9*** | ***142.62*** | ***8*** | ***11*** | ***11*** | ***118007*** | ***Cytosolic carboxypeptidase 1 (Fragment)*** |
| ***Arthrobacter woluwensis*** | ***A0A2T3BWF6*** | ***53.50*** | ***4*** | ***3*** | ***3*** | ***46603*** | ***D-alanyl-D-alanine carboxypeptidase/D-alanyl-D-alanine-endopeptidase*** |
| ***Loxosceles similis*** | ***A0A1B2ASB0*** | ***44.67*** | ***12*** | ***4*** | ***0*** | ***34989*** | ***Loxtox protein*** |
| ***Loxosceles similis*** | ***A0A1B2ASB2*** | ***48.33*** | ***12*** | ***4*** | ***1*** | ***34954*** | ***Loxtox protein*** |
| ***Stegodyphus mimosarum*** | ***A0A087UE58*** | ***68.47*** | ***6*** | ***5*** | ***5*** | ***52160*** | ***Lysosomal Pro-X carboxypeptidase (Fragment)*** |
| ***Stegodyphus mimosarum*** | ***A0A087TGU3*** | ***90.70*** | ***7*** | ***2*** | ***1*** | ***25458*** | ***Metalloendopeptidase (Fragment)*** |
| ***Stegodyphus mimosarum*** | ***A0A087T4J3*** | ***109.52*** | ***24*** | ***12*** | ***12*** | ***47204*** | ***Metalloendopeptidase (Fragment)*** |
| ***Araneus ventricosus*** | ***A0A4Y2TGT4*** | ***69.25*** | ***3*** | ***7*** | ***7*** | ***174038*** | ***Peptidase S1 domain-containing protein*** |
| ***Araneus ventricosus*** | ***A0A4Y2HAV4*** | ***54.11*** | ***17*** | ***2*** | ***1*** | ***6190*** | ***Peptidase S1 domain-containing protein (Fragment)*** |
| ***Ornithodoros erraticus*** | ***A0A293LI19*** | ***110.54*** | ***16*** | ***5*** | ***1*** | ***25697*** | ***Peroxiredoxin 1*** |
| ***Stegodyphus mimosarum*** | ***A0A087T0M9*** | ***108.76*** | ***19*** | ***5*** | ***1*** | ***21774*** | ***Peroxiredoxin 1 (Fragment)*** |
| ***Stegodyphus mimosarum*** | ***A0A087TJI3*** | ***83.05*** | ***13*** | ***11*** | ***11*** | ***65290*** | ***Phosphoinositide phospholipase C (Fragment)*** |
| ***Loxosceles gaucho*** | ***Q4VDB5*** | ***44.66*** | ***11*** | ***5*** | ***1*** | ***31282*** | ***Phospholipase D LgSicTox-alphaIA1*** |
| ***Loxosceles intermedia*** | ***Q2XQ09*** | ***45.66*** | ***10*** | ***3*** | ***3*** | ***34314*** | ***Phospholipase D LiSicTox-betaIA1i*** |
| ***Araneus ventricosus*** | ***A0A4Y2BXY3*** | ***63.73*** | ***4*** | ***3*** | ***3*** | ***51427*** | ***Plasma kallikrein*** |
| ***Araneus ventricosus*** | ***A0A4Y2EQ05*** | ***50.15*** | ***6*** | ***3*** | ***3*** | ***37640*** | ***Plasminogen*** |
| ***Ornithodoros moubata*** | ***A0A1Z5L4J2*** | ***127.28*** | ***29*** | ***7*** | ***2*** | ***21886*** | ***Proteasome subunit alpha type (Fragment)*** |
| ***Araneus ventricosus*** | ***A0A4Y2AYW9*** | ***63.14*** | ***7*** | ***8*** | ***5*** | ***90803*** | ***Protein masquerade*** |
| ***Araneus ventricosus*** | ***A0A4Y2BLE7*** | ***98.43*** | ***10*** | ***4*** | ***4*** | ***37684*** | ***Protein masquerade*** |
| ***Amblyomma parvum*** | ***A0A023FX30*** | ***129.93*** | ***18*** | ***7*** | ***7*** | ***41824*** | ***Putative actin*** |
| ***Amblyomma cajennense*** | ***A0A023FII7*** | ***57.67*** | ***8*** | ***2*** | ***1*** | ***32683*** | ***Putative aldehyde dehydrogenase (Fragment)*** |
| ***Stegodyphus mimosarum*** | ***A0A087U2G5*** | ***58.21*** | ***7*** | ***4*** | ***4*** | ***55863*** | ***Putative carboxypeptidase PM20D1 (Fragment)*** |
| ***Stegodyphus mimosarum*** | ***A0A087UKJ5*** | ***55.37*** | ***21*** | ***7*** | ***7*** | ***27491*** | ***Putative chitinase 3 (Fragment)*** |
| ***Parasteatoda tepidariorum*** | ***A0A2L2Y3P1*** | ***86.84*** | ***17*** | ***4*** | ***4*** | ***29243*** | ***Putative multifunctional chaperone ixodes scapularis multifunctional chaperone*** |
| ***Stegodyphus mimosarum*** | ***A0A087TDX4*** | ***72.12*** | ***4*** | ***3*** | ***2*** | ***51686*** | ***Retinal dehydrogenase 1 (Fragment)*** |
| ***Araneus ventricosus*** | ***A0A4Y2I2Z4*** | ***85.70*** | ***16*** | ***10*** | ***10*** | ***43209*** | ***Serine proteinase stubble*** |
| ***Leptotrombidium deliense*** | ***A0A443RFT3*** | ***43.12*** | ***14*** | ***1*** | ***1*** | ***10168*** | ***Small ubiquitin-related modifier (Fragment)*** |
| ***Loxosceles sp. Gran Canaria 1*** | ***A0A0E3X297*** | ***46.91*** | ***10*** | ***4*** | ***1*** | ***30425*** | ***Sphingomyelinase D-like protein (Fragment)*** |
| ***Ixodes ricinus*** | ***A0A0K8R510*** | ***117.07*** | ***42*** | ***7*** | ***7*** | ***11838*** | ***Thioredoxin*** |
| ***Araneus ventricosus*** | ***A0A4Y2D3V5*** | ***83.41*** | ***9*** | ***10*** | ***10*** | ***91221*** | ***Transmembrane protease serine 9*** |
| ***Ornithodoros erraticus*** | ***A0A293LBP9*** | ***69.58*** | ***15*** | ***1*** | ***1*** | ***13402*** | ***Tubulin beta chain (Fragment)*** |
| ***Clonorchis sinensis*** | ***A0A3R7CDS6*** | ***70.93*** | ***6*** | ***3*** | ***3*** | ***31527*** | ***U21-ctenitoxin-Pn1a*** |
| ***Araneus ventricosus*** | ***A0A4Y2FIQ0*** | ***68.99*** | ***12*** | ***3*** | ***3*** | ***20349*** | ***U24-ctenitoxin-Pn1a*** |

Proteins identified were filtered with a cutoff above 40 (-10logP), and databases used were: *Loxosceles* genera, *Araneus ventricosus*, *Stegodyphus mimosarum*, *Phoneutria nigriventer* and Ctenitoxins; all databases were downloaded from Uniprot databases.

**Supplementary Table S3 - Proteins similarities between proteomes midgut/digestive fluid spiders analysis.**

| **Protein class** | ***Uloboru*s sp.** | ***S. mimosarum*** | ***A. geniculata*** | ***N. cruentata*** |
| --- | --- | --- | --- | --- |
| **Endopeptidases** | **Cathepsin L, O** | **Cathepsin B, D, L** | **Cathepsin B, D, L** | **Cathepsin B, F, L** |
|  | **Astacins** | **Astacins** | **Astacins** | **Astacins** |
|  | **Serine peptidase** | **CUB-LDL trypsin/Serine peptidase** | **Serine peptidase** | **CUB-LDL trypsin** |
| **Exopeptidases** | **Metallo/Serine carboxypeptidase** | **Carboxypeptidase B1** | **Carboxypeptidase A1, B1** | **Carboxypeptidase B1, B2, E** |
| **Carbohydrases** | **Alpha-1,2-mannosidase** | **Alpha-mannosidase** | **---------------** | **Lysosomal alpha-mannosidase** |
|  | **Putative chitinase** | **Probable chitinase 3** | **Putative chitinase** | **Chitinase, probable chitinase 2, 3** |
|  | **Alpha-amylase** | **Alpha-amylase** | **---------------** | **Pancreatic alpha-amylase** |
| **Lipases** | **Phosphoinositide phospholipase C** | **Phospholipase A2, B, C** | **Phospholipase B** | **Phospholipase A2, B** |
| **Toxins** | **Phospholipase D LgSicTox-alphaA1** | **Dermonecrotic toxin LiSicTox-betalA1ii** | **Dermonecrotic toxin LiSicTox-betalD1** | **Sphingomyelinase D-like protein** |
|  | **Sphingomyelinase D-like protein** |  |  |  |
|  | **U-24 Ctenitoxin** | **---------------** | **---------------** | **U-24 Ctenitoxin** |

**Proteins classes from proteomes of the midgut, digestive fluid, and abdomen similarities between *Uloborus sp*., *S. mimosarum*, *A. geniculata*  and, *N. cruentata*.**

**Supplementary Figure S4 -** Maximum Likelihood phylogenetic tree from SMaseD sequences. A Maximum Likelihood phylogenetic tree analysis by IQ Tree software, composed of midgut, venom gland, and salivary gland SMaseD sequences from spiders, scorpions, ticks, and mites. Bootstrap test of phylogeny was applied with 1000 resampling. All sequences are listed in Supplementary 1. Abbreviations: Unsp - Unspecified.

**
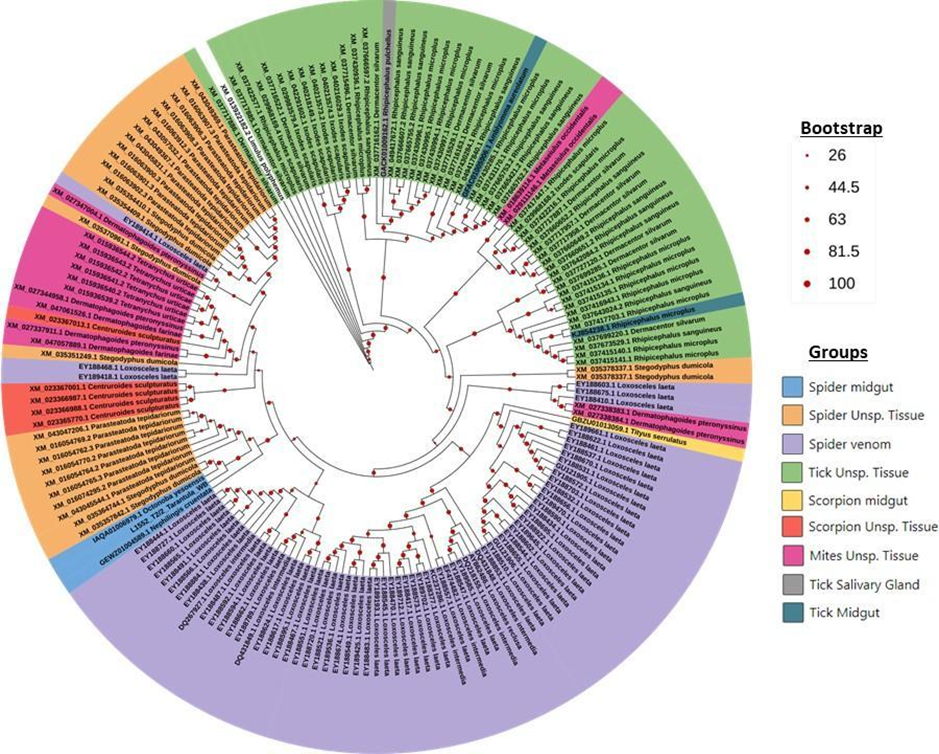
**

**Supplementary Table S5 -** Sequences list used in multiple sequences alignment and phylogenetic trees.

| **Sequences access number** | | | | | | | |
| --- | --- | --- | --- | --- | --- | --- | --- |
| 1.XM_013922182.2: Limulus polyphemus | 27.EY188660.1 Loxosceles laeta | 53.EY189193.1 Loxosceles laeta | 79.XM_027344958.1 Dermatophagoides pteronyssinus | 105.XM_037660649.2 Rhipicephalus sanguineus | 131.XM_016063902.3 Parasteatoda tepidariorum | 157.XM_037422216.1 Rhipicephalus microplus |  |
| 2.L1552_T2/2_Tarantula_WB | 28.EY188656.1 Loxosceles laeta | 54.EY188789.1 Loxosceles laeta | 80.XM_027338384.1 Dermatophagoides pteronyssinus | 106.XM_037660652.2 Rhipicephalus sanguineus | 132.XM_016063901.3 Parasteatoda tepidariorum | 158.XM_037417703.1 Rhipicephalus microplus |  |
| 3.gb\|GEWZ01004589.1 Nephilingis cruentata | 29.EY188655.1 Loxosceles laeta | 55.EY188727.1 Loxosceles laeta | 81.XM_027338383.1 Dermatophagoides pteronyssinus | 107.XM_037643024.2 Rhipicephalus sanguineus | 133.XM_016063900.3 Parasteatoda tepidariorum | 159.XM_037417447.1 Rhipicephalus microplus |  |
| 4.GBZU01013059.1 Tityus serrulatus | 30.EY188853.1 Loxosceles laeta | 56.EY188720.1 Loxosceles laeta | 82.XM_027337911.1 Dermatophagoides pteronyssinus | 108.XM_037642084.2 Rhipicephalus sanguineus | 134.XM_043045544.1 Parasteatoda tepidariorum | 160.XM_037416943.1 Rhipicephalus microplus |  |
| 5.IAQA01006979.1 Octonoba yesoensis | 31.EY189661.1 Loxosceles laeta | 57.EY188624.1 Loxosceles laeta | 83.XM_015936544.2 Tetranychus urticae | 109.XM_037673529.1 Rhipicephalus sanguineus | 135.XM_040216029.3 Ixodes scapularis | 161.XM_037415141.1 Rhipicephalus microplus |  |
| 6.AY862486.1 Loxosceles reclusa | 32.EY189425.1 Loxosceles laeta | 58.EY188622.1 Loxosceles laeta | 84.XM_015936543.2 Tetranychus urticae | 110.DQ267927.1 Loxosceles intermedia | 136.XM_029968169.4 Ixodes scapularis | 162.XM_037415140.1 Rhipicephalus microplus |  |
| 7.EY188922.1 Loxosceles laeta | 33.EY189418.1 Loxosceles laeta | 59.EY188618.1 Loxosceles laeta | 85.XM_015936542.2 Tetranychus urticae | 111.EF474482.1 Loxosceles intermedia | 137.XM_042291602.1 Ixodes scapularis | 163.XM_037415136.1 Rhipicephalus microplus |  |
| 8.EY189712.1 Loxosceles laeta | 34.EY189414.1 Loxosceles laeta | 60.EY188617.1 Loxosceles laeta | 86.XM_015936541.2 Tetranychus urticae | 112.DQ431849.1 Loxosceles intermedia | 138.XM_029983579.4 Ixodes scapularis | 164.XM_037415135.1 Rhipicephalus microplus |  |
| 9.EY189702.1 Loxosceles laeta | 35.EY189645.1 Loxosceles laeta | 61.EY188603.1 Loxosceles laeta | 87.XM_015936540.2 Tetranychus urticae | 113.DQ431848.1 Loxosceles intermedia | 139.XM_040213574.3 Ixodes scapularis | 165.XM_037415134.1 Rhipicephalus microplus |  |
| 10.EY188884.1 Loxosceles laeta | 36.EY188377.1 Loxosceles laeta | 62.EY188602.1 Loxosceles laeta | 88.XM_015936539.2 Tetranychus urticae | 114.XM_047061526.1 Dermatophagoides farina | 140.XM_040213573.2 Ixodes scapularis | 166.XM_037431170.1 Rhipicephalus microplus |  |
| 11.EY188470.1 Loxosceles laeta | 37.EY188557.1 Loxosceles laeta | 63.EY188595.1 Loxosceles laeta | 89.XM_023367013.1 Centruroides sculpturatus | 115.XM_047057889.1 Dermatophagoides farina | 141.XM_040218149.1 Ixodes scapularis | 167.XM_037430997.1 Rhipicephalus microplus |  |
| 12.EY188468.1 Loxosceles laeta | 38.EY188551.1 Loxosceles laeta | 64.EY188594.1 Loxosceles laeta | 90.XM_023367001.1 Centruroides sculpturatus | 116.XM_043057532.1 Parasteatoda tepidariorum | 142.XM_029968168.2 Ixodes scapularis | 168.XM_037430996.1 Rhipicephalus microplus |  |
| 13.EY188467.1 Loxosceles laeta | 39.EY188549.1 Loxosceles laeta | 65.EY188592.1 Loxosceles laeta | 91.XM_023366988.1 Centruroides sculpturatus | 117.XM_016074295.2 Parasteatoda tepidariorum | 143.XM_037717958.1 Dermacentor silvarum | 169.XM_037430995.1 Rhipicephalus microplus |  |
| 14.EY188461.1 Loxosceles laeta | 40.EY188547.1 Loxosceles laeta | 66.EY188590.1 Loxosceles laeta | 92.XM_023366987.1 Centruroides sculpturatus | 118.XM_043049369.1 Parasteatoda tepidariorum | 144.XM_037717957.1 Dermacentor silvarum | 170.XM_037430994.1 Rhipicephalus microplus |  |
| 15.EY188444.1 Loxosceles laeta | 41.EY188545.1 Loxosceles laeta | 67.EY188573.1 Loxosceles laeta | 93.XM_023365770.1 Centruroides sculpturatus | 119.XM_043049367.1 Parasteatoda tepidariorum | 145.XM_037717887.1 Dermacentor silvarum | 171.XM_037430936.1 Rhipicephalus microplus |  |
| 16.EY188438.1 Loxosceles laeta | 42.EY188544.1 Loxosceles laeta | 68.EY188572.1 Loxosceles laeta | 94.KC237286.1 Loxosceles intermedia | 120.XM_043047206.1 Parasteatoda tepidariorum | 146.XM_037717886.1 Dermacentor silvarum | 172.XM_037430285.1 Rhipicephalus microplus |  |
| 17.EY188434.1 Loxosceles laeta | 43.EY188537.1 Loxosceles laeta | 69.EY188566.1 Loxosceles laeta | 95.DQ218155.1 Loxosceles intermedia | 121.XM_016054770.2 Parasteatoda tepidariorum | 147.XM_037717885.1 Dermacentor silvarum | 173.XM_037430284.1 Rhipicephalus microplus |  |
| 18.EY188417.1 Loxosceles laeta | 44.EY188532.1 Loxosceles laeta | 70.EY188565.1 Loxosceles laeta | 96.XM_037665752.2 Rhipicephalus sanguineus | 122.XM_016054769.2 Parasteatoda tepidariorum | 148.XM_037716522.1 Dermacentor silvarum | 174.XM_035370961.1 Stegodyphus dumicola |  |
| 19.EY188410.1 Loxosceles laeta | 45.EY188531.1 Loxosceles laeta | 71.KJ854238.1 Rhipicephalus microplus | 97.XM_049417840.1 Rhipicephalus sanguineus | 123.XM_016054765.3 Parasteatoda tepidariorum | 149.XM_037716163.1 Dermacentor silvarum | 175.XM_035364744.1 Stegodyphus dumicola |  |
| 20.EY189470.1 Loxosceles laeta | 46.EY188521.1 Loxosceles laeta | 72.GFAC01005906.1 Amblyomma aureolatum | 98.XM_037666597.2 Rhipicephalus sanguineus | 124.XM_016054764.2 Parasteatoda tepidariorum | 150.XM_037716162.1 Dermacentor silvarum | 176.XM_035357842.1 Stegodyphus dumicola |  |
| 21.EY188703.1 Loxosceles laeta | 47.EY188510.1 Loxosceles laeta | 73.GACK01009162.1 Rhipicephalus pulchellus | 99.XM_049417672.1 Rhipicephalus sanguineus | 125.XM_016054762.3 Parasteatoda tepidariorum | 151.XM_037715529.1 Dermacentor silvarum | 177.XM_035354410.1 Stegodyphus dumicola |  |
| 22.EY188695.1 Loxosceles laeta | 48.EY188491.1 Loxosceles laeta | 74.GU121906.1 Loxosceles laeta | 100.XM_037665755.2 Rhipicephalus sanguineus | 126.XM_016063912.3 Parasteatoda tepidariorum | 152.XM_037715496.1 Dermacentor silvarum | 178.XM_035354409.1 Stegodyphus dumicola |  |
| 23.EY188675.1 Loxosceles laeta | 49.EY188487.1 Loxosceles laeta | 75.GU121905.1 Loxosceles laeta | 101.XM_037665753.2 Rhipicephalus sanguineus | 127.XM_016063907.3 Parasteatoda tepidariorum | 153.XM_037699285.1 Dermacentor silvarum | 179.XM_035378337.1 Stegodyphus dumicola |  |
| 24.EY188674.1 Loxosceles laeta | 50.EY188483.1 Loxosceles laeta | 76.XM_029111146.1 Metaseiulus occidentalis | 102.XM_037666607.2 Rhipicephalus sanguineus | 128.XM_016063908.3 Parasteatoda tepidariorum | 154.XM_037699220.1 Dermacentor silvarum | 180.XM_035351249.1 Stegodyphus dumicola |  |
| 25.EY188670.1 Loxosceles laeta | 51.EY189585.1 Loxosceles laeta | 77.XM_018639114.1 Metaseiulus occidentalis | 103.XM_037665421.2 Rhipicephalus sanguineus | 129.XM_016063906.3 Parasteatoda tepidariorum | 155.XM_037727120.1 Dermacentor silvarum |  |  |
| 26.EY188662.1 Loxosceles laeta | 52.EY189536.1 Loxosceles laeta | 78.XM_027347004.1 Dermatophagoides pteronyssinus | 104.XM_037660651.2 Rhipicephalus sanguineus | 130.XM_043045831.1 Parasteatoda tepidariorum | 156.XM_037422577.1 Rhipicephalus microplus |  |  |
